# Deconvolution of Human Brain Cell Type Transcriptomes Unraveled Microglia-Specific Potential Biomarkers

**DOI:** 10.3389/fneur.2018.00266

**Published:** 2018-04-26

**Authors:** R. Ayana, Shailja Singh, Soumya Pati

**Affiliations:** ^1^Department of Life Sciences, School of Natural Sciences, Shiv Nadar University, Lucknow, India; ^2^Special Centre for Molecular Medicine, Jawaharlal Nehru University, New Delhi, India

**Keywords:** *SPHK1*, age, microglia stem cell-like progenitors, *PTX3*, transcriptomic analysis, amygdala, hippocampus, striatum

## Abstract

Microglial cells form a context-dependent network of brain immunoeffector cells. Despite their indispensable roles, unresolved questions exist around biomarker discovery relevant to their cellular localization, self-renewing potential, and brain developmental dynamics. To resolve the existent gap in the annotation of candidate biomarkers, we conducted a meta-analysis of brain cells using available high-throughput data sets for deciphering microglia-specific expression profiles. We have identified 3,290 significant genes specific to microglia and further selected the top 20 dysregulated genes on the basis of *p*-value and log_2_FC. To this list, we added 7 known microglia-specific markers making the candidate list comprising 27 genes for further downstream analyses. Next, we established a connectome of these potential markers with their putative protein partners, which demonstrated strong associations of upregulated genes like Dedicator of cytokinesis 2 (*DOCK2*) with early/mature microglial markers such as Sphingosine kinase 1 (*SPHK1*), *CD68*, and *CD45*. To elucidate their respective brain anatomical location, we deconvoluted the BrainSpan Atlas expression data. This analysis showed high expression of the majority of candidate genes in microglia-dense regions (Amygdala, Hippocampus, Striatum) in the postnatal brain. Furthermore, to decipher their localized expression across brain ages, we constructed a developmental dynamics map (DDM) comprising extensive gene expression profiles throughout prenatal to postnatal stages, which resulted in the discovery of novel microglia-specific gene signatures. One of the interesting readout from DDM is that all the microglia-dense regions exhibit dynamic regulation of few genes at 37 post conception week (pcw), the transition period between pre- and postnatal stages. To validate these findings and correlate them as potential biomarkers, we analyzed the expression of corresponding proteins in hESC-derived human microglia precursors. The cultured microglial precursors showed expression of Pentraxin 3 (*PTX3*) and *SPHK1* as well as several known markers like *CD68*, Allograft inflammatory factor 1 (*AIF1*/*IBA1*). In summary, this study has furnished critical insights into microglia dynamics across human brain ages and cataloged potential transcriptomic fingerprints that can be further exploited for designing novel neurotherapeutics.

## Introduction

The complex human brain is composed of neurons embedded in a framework of glial cells (astrocytes, oligodendrocytes, and microglia) and blood vessels. Microglial cells, the third and highly motile type of glial cell populating the brain were first identified and simultaneously reported by F. Robertson and F. Nissl, who first gave them the name “Staebchenzellen” based on their rod-like nuclei shape (1, 2). Later, these cells were renamed as “microglia” by Pio del Rio-Hortega, who further characterized and delimited them from other glial types (3). It is noteworthy that the primary role of microglia is to dynamically monitor CNS-invading threats. Studies have proven that microglia play critical functions like maintenance of neuronal development by continuous patrolling of active neurons, scavenging dead neurons and dendritic spine pruning under normal physiological conditions (4–6). Abnormal microglial activities can either under-prune or over-trim the dendritic spines leading to progressive neurodevelopmental defects (5). Besides this, these brain scavengers can play multidimensional roles in maintaining brain homeostasis. One of the newly discovered roles suggests that microglia regulates the number of healthy neural precursor cells (NPCs) in adult brain (7, 8). First, microglia control the number of NPCs by selectively colonizing near proliferative NPC zones and phagocytizing the neural precursors *in utero*, and second, the microglial activity gets regulated in the brain by NPC-derived chemokines, such as *VEGF*, as shown by increased microglial density surrounding NPC pool (9, 10). Notably, this NPC-microglia crosstalk is most active during neuroinflammation, as microglia is one of the primary innate immunoeffector cells (10, 11). They usually accumulate around degenerating neurons, leading to the obvious misconception of causing detrimental effects. Albeit monumental efforts made toward understanding microglia dynamics in the developing brain, knowledge regarding their cellular localization, self-renewal/expansion property, and other stage-specific roles is still in its infancy.

Origin and continual existence of microglia throughout human brain span are being currently investigated. Interestingly, microglial ontogeny describes three lines of opinions. This includes its emergence either during the embryonic day (E7.0–E10.5) from yolk sac (YS) (12–15) or their infiltration during diseased conditions from bone-marrow (16). There is also a third view which suggests that microglia is of neuroectodermal origin, derived either from glioblasts or from the germinal matrix (17). In a recent study, Tay et al. established an *in vivo* fate mapping system and studied proliferation of microglia in the healthy and diseased brain which demonstrated a context-dependent growth pattern of microglia under normal physiological conditions and selective clonal expansion during disease states (18).

**Figure 1:**
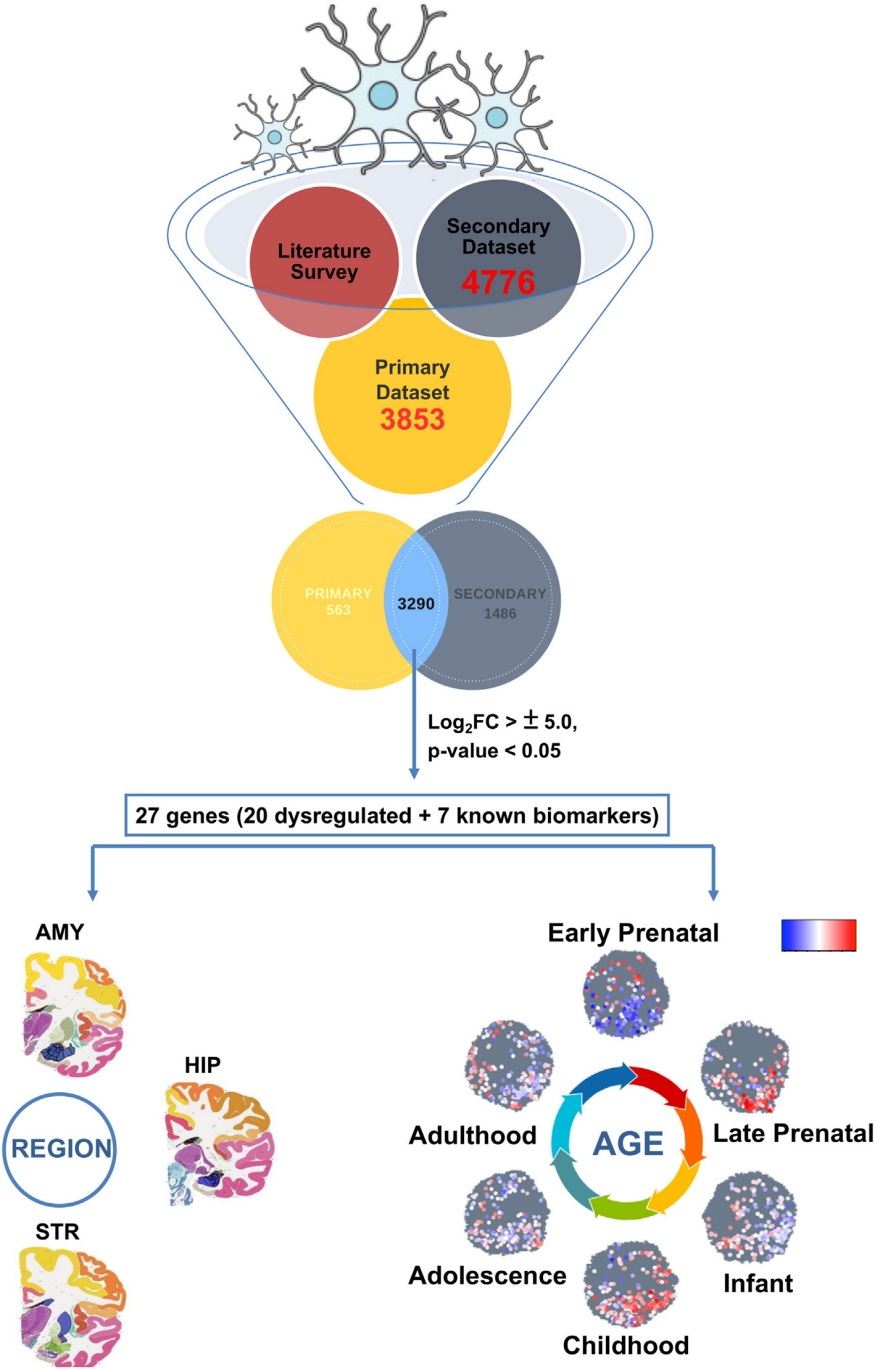
Primary strategy (*in silico* approach) employed to track microglial gene expression dynamics within the developing human brain.

The burning questions of the hour deal with life-long inhabitation and maintenance of microglia in the human brain. Answers to these will shed light underlying disease onset and manifestations in several neurological diseases including Alzheimer’s disease and Parkinson’s disease (AD and PD). One of the challenges in microglial biology is to track their expression and activity throughout the brain development (fetal to adult and aged brain stages). Till date, hardly any study exists that has shed light on *in silico* or *in vivo* tracking of the microglial population (19). Specifically, microglial age and variance over brain regions can be the guidance cue to understanding onset and progression of neurodegenerative disorders (20–23). To track microglia-specific biomarker expression, their localization, and generation of a regulatory dynamic map, this study presents an integrated meta-analysis approach wherein, we analyzed the differentially expressed microglia-specific gene subsets using available data repositories. This led to a unique catalog of genes (27) which are dynamically/consistently expressed in the microglial population during different stages of brain development. For the first time, we have employed a novel strategy for *in silico* monitoring of microglial gene signatures to decipher their developmental dynamics map (DDM). Interestingly, protein–protein interaction network analysis revealed six different protein clusters, which exposed primary interacting partners like Tissue factor (coagulation) protein family, Retinoid X receptor gamma signaling linked *MGST* protein family, ATP-dependent protein family, and hippocampal BMP signaling molecules like *BMP4*. Furthermore, region specificity of the above candidates was evaluated using Allen Brain Atlas’s (ABA) BrainSpan which revealed localized expression of several genes in microglia-centric regions. Finally, to validate the emergence of early/mature microglial sig-natures *in vitro*, we induced hESC-derived microglial differentiation using an in-house protocol. In conclusion, the study has validated the expression of stage-specific markers as well as presented an updated catalog of potential age-specific microglial biomarkers, which can be used as future prognostic biomarkers. The overall strategy adopted for *in silico* analyses has been compiled (Figure 1).

## Materials and Methods

### Literature Survey

We utilized repositories including NCBI Pubmed and HighWirePress to understand the prior literature linked to microglia biology. Other high-throughput data repositories like NCBI’s Gene Expression Omnibus (GEO) and Sequence Read Archive (SRA) were scanned for biological sequence data specific to transcriptomics of human microglia.

### High-Throughput Data Analysis

First, using available RNA-sequencing data set linked to microglia transcriptomics (24), we delineated the brain cell-type specific expression in accordance with different brain cell types namely, microglia, astrocytes, endothelium, oligodendrocytes, and neurons. Specifically, we evaluated the differential expression between Rest of the Brain Cells comprising astrocytes, endothelial cells, oligodendrocytes and neurons (ROBC), and microglial cell type. Log_2_FoldChange (FC) values threshold of ±1.5 was considered with a *p*-value significance of <0.05. This formed our *primary* data set comprising microglia cell type-specific genes. Our *secondary* data set was utilized to gauge the correlation of differential expression between brain cell types and differential expression of induced pluripotent stem cells (iPSCs) versus derived microglia. This data series deposited in GEO database contains human microglia-specific microarray Affy HuGene-2_0-st data (GSE78115) (25). Specifically, we considered the 12 samples (3 replicates each) derived from 2 different cell lines NCRM-5 and iNC-01 of iPSC origin. Using OLIGO package of R Bioconductor, we performed data analysis (including data normalization) to assess the differential expression pattern specific to human microglia with respect to iPSCs. The OLIGO package is freely available through Bioconductor website (26). Differential expression cutoff of ±1.5 log_2_FC and *p*-value <0.05 was considered significant in all cases. The differentially expressed genes from both primary and secondary human microglia sample sets were combined to ensure minimal data loss. This integration yielded a common pool of microglial specific genes (3,290), which was further used for downstream analyses. Pearson correlation coefficient was calculated for the integrated gene list (3,290) using R. We further analyzed the expression patterning between the *primary* and *secondary* data sets using the top 20 (out of 3,290) up and downregulated genes, which were shortlisted on the basis of *p*-value and log2FC values below 0.01 and above ±5.0, respectively. To this list, we also added a list of seven known biomarker genes specific to microglia for understanding their development expression dynamics. Taken together, the total list used for brain age and region-specific dynamics consisted of 27 genes.

### Protein Classification and Pathway Analysis

We utilized Protein ANalysis THrough Evolutionary Relationships classification system and mapped the total differentially expressed list of 3,290 genes to various protein classes for elucidating their functional significance (27). For pathway analysis, we inputted the same gene list to Consensus PathDB-human to identify their key associative pathways (28). The Bonferroni corrected *p*-value cutoff <0.01 was applied for both analyses.

### Gene Set Enrichment Analysis

In order to ascertain significant gene sets in our differentially expressed gene list, we implemented Gene Set Enrichment analysis using the Broad Institute GSEA v2.07 software,^1^ the molecular signatures database,^2^ and the C5: GO gene sets database, comprised of 1,454 gene sets named by genes and respective GO terms.^3^

### Protein–Protein Interaction Network Analysis

The differentially expressed gene list (3290) was mapped to STRING for whole proteome network analysis. Furthermore, the shortlisted microglia-specific gene list (27) was also analyzed for protein partner associations using STRING (29). Primarily, using all the top upregulated and downregulated protein clusters, a single protein–protein network was constructed. A stringent cut-off of 0.7 for edge/interaction score was considered for building the primary protein–protein interaction network without clustering and no more than 20 primary interactor proteins. This combined confidence score is calculated on the basis of various parameters including phylogenetic co-occurrence, gene fusion, homology, co-expression, experimentally determined interaction, and neighborhood on any chromosome. The graph was primarily constructed according to interaction/edge score while specifying the source and target nodes. All networks were visualized using Cytoscape 3.4.0 (30).

### Brain Region Expression Mapping

The Allen Brain Atlas contains an extensive catalog of human brain expression data (Microarray and RNA-Seq) with region specificity (31, 32). We collected the raw data of the developmental transcriptome from Allen Brain Atlas and segregated the data into different ages from prenatal to postnatal (adult) brain (31). Here, we mapped the top gene set of 27 genes onto all the prenatal and postnatal brain regions, with a special emphasis on human microglia-enriched regions like Hippocampus, Striatum, and Amygdala. Other than these genes, we also show the expression of known microglial marker genes like *TMEM119, P2RY12*, Phospholipase D4 (*PLD4*), *AIF1, PTPRC1*, Sphingosine kinase 1 (*SPHK1*), *PTX3*, and G protein-coupled receptor 34 (*GPR34*).

### Age-Wise Dynamics

To assess the microglia dynamics throughout the age (pre- and postnatal) of a human being, we utilized the human developmental transcriptome data (BrainSpan) deposited in the Allen Brain database (33, 34). Replicate samples for each developmental stage (prenatal or postnatal) were considered for analysis. The age groups ranged from prenatal stages, including 14th postconception week (pcw), 22 pcw to 40 years of age. Specifically, for each microglia-enriched region of the brain (hippocampus, striatum, and amygdala), we constructed individual graphs for age-wise DDM of potential and known microglia-specific markers which included shortlisted gene set of 27, containing 20 (*p*-value < 0.01, log_2_FC > ±5.0) and 7 known markers. Following this, correlation analysis was performed using the same gene list as input to find correlations among the human ages spanning prenatal stages like 12 pcw, 37 pcw to postnatal stages, such as 1, 15–19, and 40 years of age. Pearson correlation matrices or correlograms were constructed and visualized using the Corrplot package of R Bioconductor suite. All graphs have been made using RStudio 1.0.153 (35).

### Culturing of Human Embryonic Stem Cells (H9)

Human embryonic stem cells (h9-hESC, NIH approved) were obtained from human embryonic stem cell core, Baylor College of Medicine. H9 clones were propagated based on feeder-free culture system, using human ESC-qualified Matrix, BD Matrigel (BD Biosciences, San Jose, CA, USA), and grown in mTeSR-1 complete media supplements (Stemcell Technologies, Vancouver, BC, Canada) at 37°C and 5% CO_2_. Clones were maintained and expanded according to manufacturer’s protocol, in accordance with NIH guidelines. Complete media was changed every day and cells were sub-cultured using Dispase (Stemcell Technologies, Vancouver, BC, Canada) every 5–6 days. During each stage of sub-culture, uniform H9 colonies were collected without any visible differentiation.

### Embryonic Body (EB) Formation

To induce the formation of EBs or neuroectodermal spheres, the H9 colonies with 70% confluency were used. To achieve neural induction, H9 colonies were incubated in neural induction me-dium [NIM; DMEM/F12: Neurobasal (1:1), 2% B27, 1% N2 (Invitrogen)] for almost 24 h and dissociated into small clumps by manual scraping. Small clumps were further grown as suspension culture with NIM in non-coated bacterial Petri dishes for a period of 4 days as exactly described by Cho et al. (36). 50% of the media were replaced in each of the EB-containing Petri dishes at a timely interval of 48 h.

### Microglial Differentiation and Generation of Microglia Precursors From hESC

To induce microglial differentiation in culture from H9 derived EBs and obtain human microglial precursors *in vitro*, we used an in-house standardized protocol (unpublished), modified based on an earlier established protocol (37). In brief, the whole process goes through a long-term culture (approximately 8 weeks) covering four major steps, (i) formation of hESC (H9) derived EBs, (ii) neuroectodermal lineage commitment, (iii) microglial differentiation, and (iv) formation of induced microglia precursors (iMPs), as suggested in the previous article by Beutner et al. (37). We have used stage-specific conditioned media supplements as suggested by Beutner et al., including differentiation media (DIFF), Insulin/Transferrin/Selenite/Fibronectin media (ITSF_n_) and N2 Media. However, we have introduced several modifications during the stage-specific progression of microglial differentiation. These include the following: (1) Usage of NIM media till 5 Days *in vitro* (DIV) for maintaining EBs and then switching them to ITSF_n_ on sixth day, (2) addition of human stem cell factor (hSCF, Peprotech, USA) and basic fibroblast growth factor (Peprotech) in culture from DIV 7 to DIV 14 with concentrations of 50 and 25 ng/ml, respectively, (3) treatment of cultures from DIV 14 to DIV 21 with hSCF, hIL3, and human granulocyte macrophage stimulating factor (hGM-CSF, Peprotech) with concentrations of 50 ng/ml for both hSCF and hIL3, and 25 ng/ml for hGM-CSF, and finally (4) addition of only hGM-CSF from DIV 21 onward till DIV 40 and furthermore.

### Immunocytochemistry/Immunofluorescence Analysis

Cells from different stages of microglia induction and differentiation were fixed for performing immunocytochemistry and immunofluorescence-based analyses using primary antibodies and fluorophore-conjugated secondary antibodies. Detailed information related to fixation, antibodies, dilution, and image analysis are listed within Data sheet 1 (Supplementary Table 1) in Supplementary Material.

### Statistical Analyses

Differential expression cutoff of ±1.5 log_2_FC and *p*-value < 0.05 was considered significant unless specified. Bonferroni corrected *p*-value cutoff = 0.01 was applied in protein classification and pathway analyses. At least triplicate donors were considered for each developmental stage (prenatal or postnatal) for age/region analyses. In case of the *in vitro* experiments, we have maintained biological replicate of *n* = 3.

## Results

### Cell-Type Specific Gene Set Enrichment Analysis of Human Microglia

**Figure 2:**
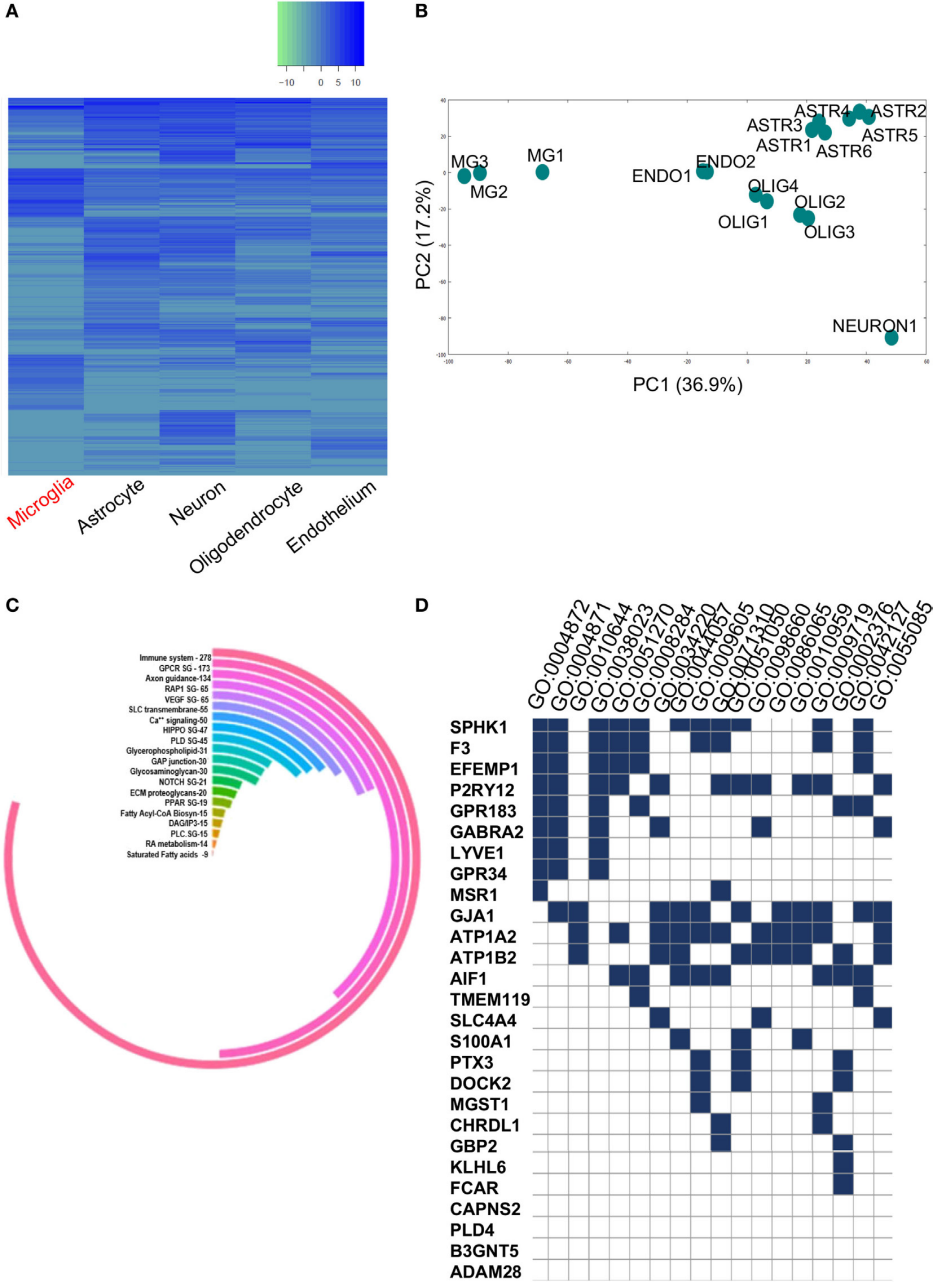
**(A)** Heatmap representing significantly expressed upregulated and downregulated gene profiles of various brain cell types. **(B)** Principal component analysis plot showing homogeneity within each cell type and diversity among the five brain cell types namely, microglia, endothelial cells, oligodendrocytes, neurons, and astrocytes. **(C)** Circular plot showing pathway analysis of total dysregulated gene list (3,290) between ROBC and microglia. **(D)** GSE Analysis showing enriched GO BP/MF terms (*p* < 0.05) in 27 shortlisted genes overlapping between primary and secondary datasets.

The complete GEO and SRA high-throughput databases were scanned to identify microglia-specific data sets. Out of this, two data sets were shortlisted for further in-depth *in silico* analyses. Initially, we analyzed the *primary* data set, i.e., the total gene set from the cell-type specific data comprised of significantly expressed genes in ROBC versus microglia (Figure 2A). A total of 3,853 genes were differentially expressed, with a range comprising 6.0 log_2_FC as the maximum expression to −9.4 log_2_FC as the minimum expression. The samples of various cellular origins were tested for homogeneity *via* 2D-Principal Component Analysis which resulted in noticeable differences in microglia sample cluster from other cell types in Principal components, PC 1 and PC 2 (Figure 2B). We specifically considered the *primary data set* to be the cell-type data to ensure elimination of non-microglia related noise in the human brain. Furthermore, we identified known biomarkers including *SPHK1, PLD4, TMEM119*, and *CD68*, expressed in the range of 2–6 FC and used them as a positive control for *in silico* tracking of microglia.

Sphingosine kinase 1 is a known biomarker for quiescent neural stem cell (qNSC) pool and the key enzyme responsible for phosphorylation of sphingosine to sphingosine-1-phosphate (*S1P*) (38). It has been also seen to play an important role in the regulation of proinflammatory cytokines in activated microglia (39). Purinergic receptor 12 (*P2RY12*) is known to suppress microglial process motility and delay in closure of blood–brain barrier (BBB) (40). *P2RY12*-mediated microglial cell activation contributes to the rapid closure of BBB small leaks, by aggregation of microglial cells at the injury site. Expression of Phospholipase D4 (*PLD4*) and its localization changes are correlated with the activation state of microglia (41, 42). This transmembrane glycoprotein localized in the endoplasmic reticulum and Golgi apparatus is primarily seen to play an important role in microglia dynamics during early postnatal brain development. Transmembrane protein 119 (*TMEM119*) is an established, reliable microglial marker that discriminates resident microglia from blood-derived macrophages in the human brain (43). A glia-derived Pentraxin 3 (*PTX3*) expressed in the microglial secretome, has been seen to modulate phagocytic functions of microglia having crucial implications in the regulation of microglial activity in brain diseases (44).

The *secondary* data set was a redundant list of total 4,776 genes which was seen to be differentially expressed among 2 human microarray data sets. This data set contains six induced microglia samples derived from human iPSCs (25). We integrated the differentially expressed microglia-specific data enriched from both *primary* and *secondary* data sets containing 3,853 and 4,776 genes, respectively. This meta-analysis yielded a common pool of microglial specific genes (3,290) which was further used for downstream analyses (Figure 1).

### Pathway Mapping and Gene Set Enrichment Analysis

The readout of gene candidates (3,290) was used to target enriched pathways. Among the candidate pathways, GPCR, Glycerophospholipid, NOTCH1, RAP1, glycosaminoglycan metabolism, VEGFR signaling, etc. were significant (Figure 2C). This analysis also demonstrated enrichment of 278 out of 3,290 genes within the immune system pathway, suggesting their potential roles in microglia functioning or immune surveillance (Figure 2C).

The list of significantly expressed genes (3,290) was used for gene/protein type-based clustering. This analysis revealed 25 crucial protein classes that majorly comprise of 10.7% enzyme regulators, followed by 9.9% nucleic acid binding proteins, and enzymes like transferases, hydrolases, transcription factors, and signaling molecules (Figure S1 in Supplementary Material). The other segments included cell junction, immunity/defense and extracellular matrix proteins containing 28, 40 and 46 proteins, respectively. These classes acted as guidance cues for the protein–protein based network analysis as well as pathway mapping and exposed many critical clusters of gene regulatory elements. We also performed GSEA on critically altered gene set of 20 dysregulated genes and 7 known microglia-specific markers to find critical GO terms like Immune System process, Receptor activity, Regulation of cell proliferation which further substantiated the data (Figure 2D; Supplementary Table 2 in Supplementary Material). The aforementioned shortlisted gene set (27) was used for further analyses.

### Correlation Analysis of Microglial Biomarkers Among the Data Sets

**Figure 3:**
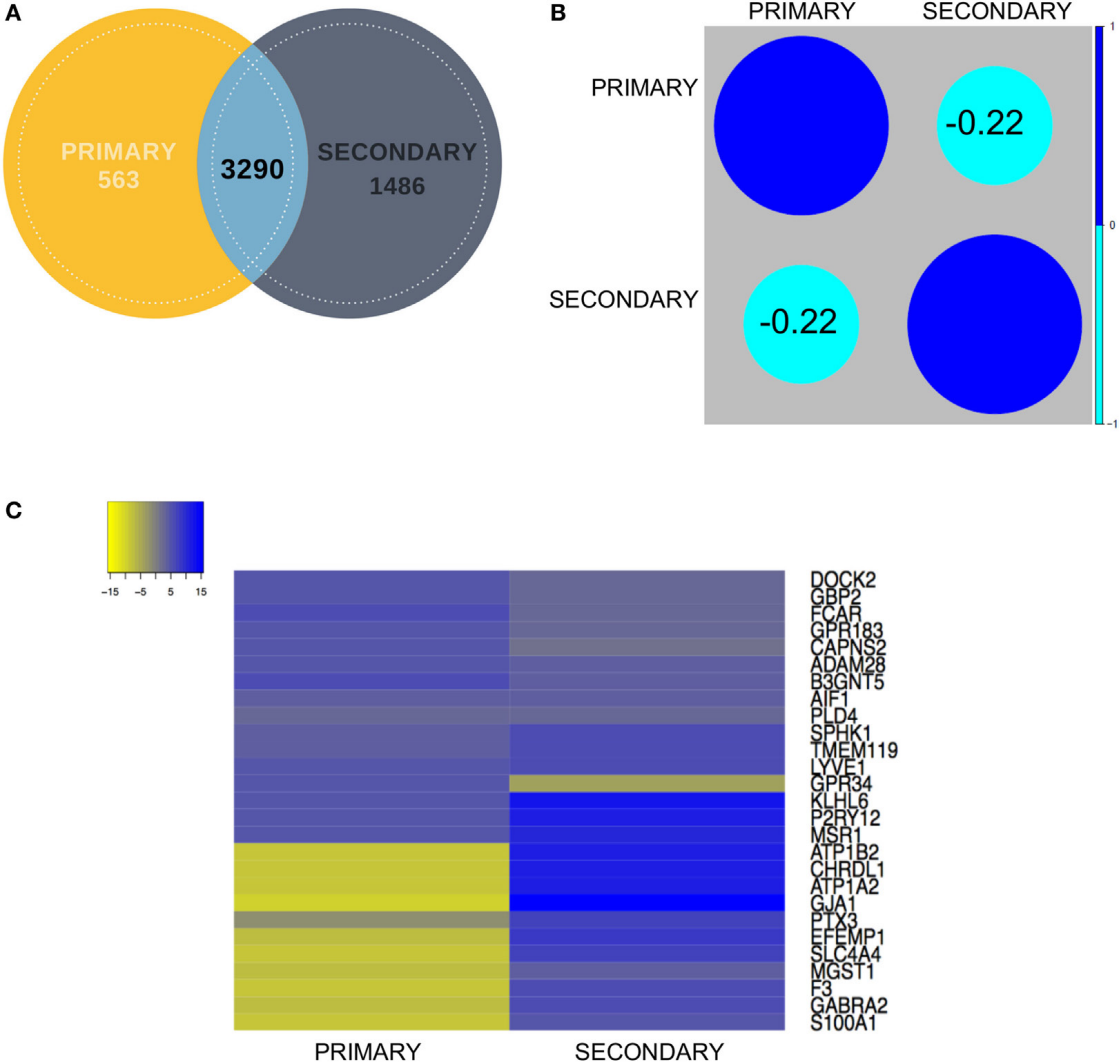
**(A)** Venn diagram showing contribution of each dataset (primary and secondary) to the overlapping gene pool. **(B)** Correlation analysis between the primary and secondary datasets showed mildly negative correlation. **(C)** Representative expression heatmap between the final shortlisted 27 genes has been elaborated in the expression map for in-depth correlation analysis.

To establish correlations among the *primary* and *secondary* data sets, we calculated the Pearson correlation coefficient for the common gene pool between the two data sets (3,290), which resulted in a value of −0.22 (Figures 3A,B). First, the primary and secondary datasets were analyzed for the similarity in expression of shortlisted gene set comprising 27 (20 novel + 7 known) genes (Figure 3C). Within the upregulated gene set, all these biomarker genes excluding *GPR34* demonstrated similarity in expression when compared to the *secondary* data set [iPSC derived microglia (iMG) data] (Figure 3B). To highlight, top upregulated genes, including Dedicator of cytokinesis 2 (*DOCK2*), Guanylate binding protein 2 (*GBP2*), A disintegrin and metalloproteinase 28 (*ADAM28*), G Protein-coupled receptor 183 (*GPR183*), Calpain small subunit 2 (*CAPNS2*), and Fc-α receptor mediator (FCAR), were found to be consistently expressed in primary and secondary data set.

Further literature mining of this top altered gene set (27) substantiated their mechanistic roles relevant to neuroinflammation and neurotoxicity. Among these, *DOCK2*, an atypical Rho guanine nucleotide exchange factor (GEF) for *RAC* and/or *CDC42* GTPases are known to regulate phagocytic activity, cytokine release, and paracrine neurotoxicity in microglia (45). In one of the microglia-mediated neuroinflammatory cascades, the *DOCK2*-*RAC1* pathway was seen to be elicited by S1P through an integrin-dependent association (46). Precisely, S1P which is the phosphorylated end-product of *SPHK1* is known to regulate immune cell trafficking upon binding to G-coupled receptors (*GPRs*) (47). Interestingly, we found fivefold expression of a novel marker *GPR183* in microglia whose role is yet to be elucidated. *DOCK2* is directly regulated by the Prostaglandin E2 receptor (*EP2*) and has also been linked with AD pathophysiology (45). Concurrent evidence involving Prostaglandin signaling in microglia also suggested that balanced induction of Peroxisome proliferator-activated receptor gamma (*PPARG)* by *LPS/IL13* or activation of *EP2* can lead to neuroprotection or death of microglia *via* calpain-dependent pathway (48). Our data also validated the elevated expression of *PPARG* as well as its downstream target calpain associated genes like *CAPNS2* in the microglial pool versus the other cell types.

Another immunoeffector molecule, *FCAR* (*CD89*) was seen to be highly expressed among the top 20 altered genes in the microglial population, which also corroborated by a recent report by Galatro et al. (49). Previous reports suggest that human microglial cells expressing truncated IRF3 show decreased expression of *GBP2*, thus proposing a possible link of this protein to *TLR3/4*-dependent neuroinflammation. Our study has shown another important marker of the microglial cell type known to be involved in CNS invasion, i.e., *ADAM28*, a member of the metalloproteinase family (50). Another enriched marker gene *N*-acetylglucosaminyltransferase *B3GNT5* was seen to be a prominent player in the expression of lactoseries sulfoglucuronylglycolipids within glycosphingolipid pathway during the prenatal brain develop-ment (51). High expression of Kelch-like family member 6 (*KLHL6*) was also detected in the microglial pool, which is supported by previous AD mice model-based studies linked it to AD pathology (52). We also found an interesting factor known as macrophage scavenger receptor 1 (*MSR1*) involved in the phagocytic machinery of microglia (53).

Contrary to this, in case of top 10 downregulated genes, the expression values for the *secondary* data set were found to be non-correlative to the primary data set (ROBC vs. microglia) (Figure 3C). Microsomal Glutathione S-Transferase 1 (*MGST1)*, a regulator of Retinoid X receptor gamma signaling during remyelination showed non-correlative expression between the two data sets (54). Genes like S100 Calcium Binding Protein A1 (*S100A1*) (55) and Gamma-aminobutyric acid receptor subunit alpha-2 (*GABRA2*) (24, 56) which are known to be highly expressed in astrocytes were also seen to be enriched in the secondary data set and repressed in the primary data set, thus justifying the need to enrich a pure microglia cell type marker-enriched population for *in silico* and *in vivo* tracking. We also detected a novel gene EGF Containing Fibulin Like Extracellular Matrix Protein 1 (*EFEMP1*), a fibulin glycoprotein family member which was found to be downregulated in ROBC versus microglia. This was earlier shown to be upregulated in malignant gliomas and age-related macular degeneration (57). Another interesting observation suggested expression of several synaptosomal and synaptic receptor genes like *SLC4A4* and *GABRA2* significantly downregulated in the primary data set in coherence with the study by Ji et al. (58). This analysis sheds light on probable roles of the top 20 genes in other brain cell types as well as exposes a filtered population of markers linked to microglia function in the developing human brain.

### Protein–Protein Interaction Network Analysis

**Figure 4:**
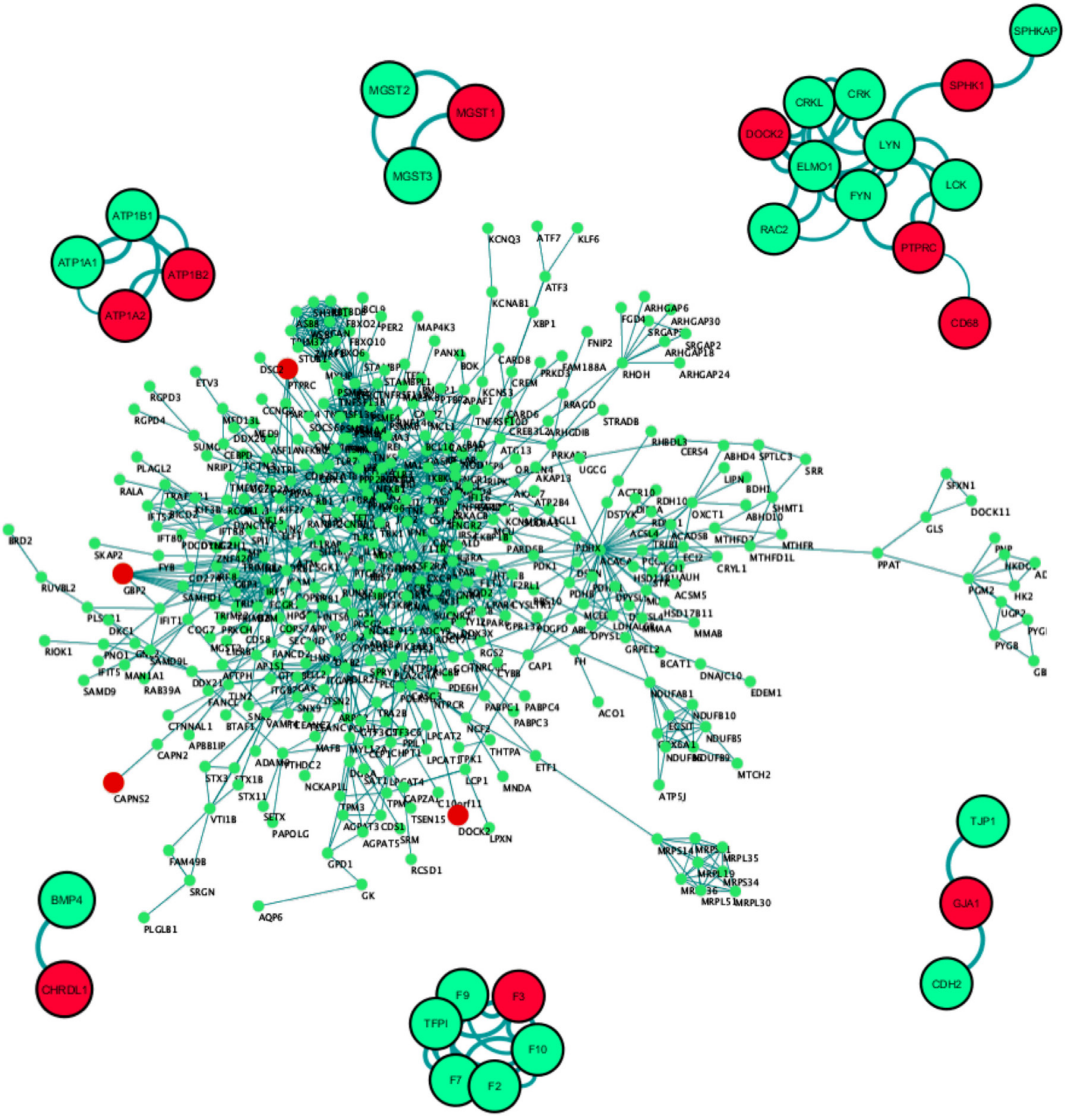
PPI network construction of dysregulated proteome coded by 3,290 genes. Only 994 out of 3,290 genes passed the threshold interaction score of 0.70. The second representative protein–protein interaction network of the top differentially expressed gene set (27 genes) showed 11 out of 27 proteins coded by differential expressed genes to be actively interacting with each other or with varied protein partners. Red: differentially expressed gene in the primary data set, green: interacting partner, edge indicates the source to target node strength. Combined interaction score > 0.70, No. of first level interactors = 20.

To develop an in-depth understanding of the microglia-specific markers and their primary neighborhood protein partners, we constructed a primary protein-protein interaction network. Out of the input list of 3,290 genes, 994 coding proteins showed interactions with a confidence score above 0.70 (Figure 4). Network statistical analysis showed the Network centralization score to be 0.07 and clustering coefficient to be 0.423 with a total number of hub nodes to be 43.

Subset PPI networks were constructed based on the top 20 differentially expressed gene list in combination with the known biomarkers. This network exposed primary neighborhood interacting partners of *DOCK2, F3, GJA1, CHRDL1, MGST1*, and *ATP1A2* (Figure 4). Among these, one of the highly upregulated genes, *DOCK2* showed multiple interacting partnerships with known biomarkers like *SPHK1, PTPRC*, and *CD68*, which is substantiated by numerous connections (edges) and their respective confidence scores (Figure 4). *DOCK2* is also seen to be connected to *SPHK1* through *LYN*, and to *PTPRC*, and *CD68* through *FYN* (Figure 4). In a second cluster, we found *GJA1* to be interacting with *CDH2* and *TJP1*. The *GJA1* mutation has been seen to cause Oculodentodigital dysplasia, an autosomal dominant disorder, with high penetrance in intra- and interfamilial phenotypes (59). In another cluster, F3 or thrombosis factor 3 was prominently associated with its own family members (Figure 4). F3 gene encodes coagulation factor 3 which is a membrane-bound glycoprotein is part of the Tissue factor family. Although no specific links were found between *F3* and brain development, it has been shown to be regulated during brain neoplasms (60) or in the innate immune cells (61, 62). Based on GSEA, correlation, and PPI analysis, we conclude that *DOCK2* acts as a molecular hub for channelized signaling in microglia-based neuroprotection, neuroinflammation, and neurological diseases, including AD and PD, Autism spectrum disorders, and schizophrenia (63). This newly discovered microglia-specific regulator is known to be associated with disease pathology of AD patients independent of COX signaling (45).

### Trends in Region-Specificity for Potential, Novel Microglia Signatures

Cellular localization of microglia is still debatable in human brain development. Notably, the prenatal human brain displays smaller brain size with a higher number of sub-regions (26), whereas the postnatal brain contains 18 developed sub-regions. Microglia population in the healthy brain is known to be residing primarily in hippocampus, amygdala, substantia nigra, and striatum. However, developmental tracking of human microglial activity in these regions is still elusive. To elucidate the region-specific dynamic regulation of microglial markers, we analyzed the distribution of the identified *in silico* candidates. Our data revealed that in the prenatal and postnatal brain regions, the expression profiles of selected dysregulated gene set (20 + 7) was dynamically regulated as seen by their variable expression across sub-regions. Understanding this variability in their expression pattern would be crucial for building the microglial dynamic map in human brain development.

**Figure 5:**
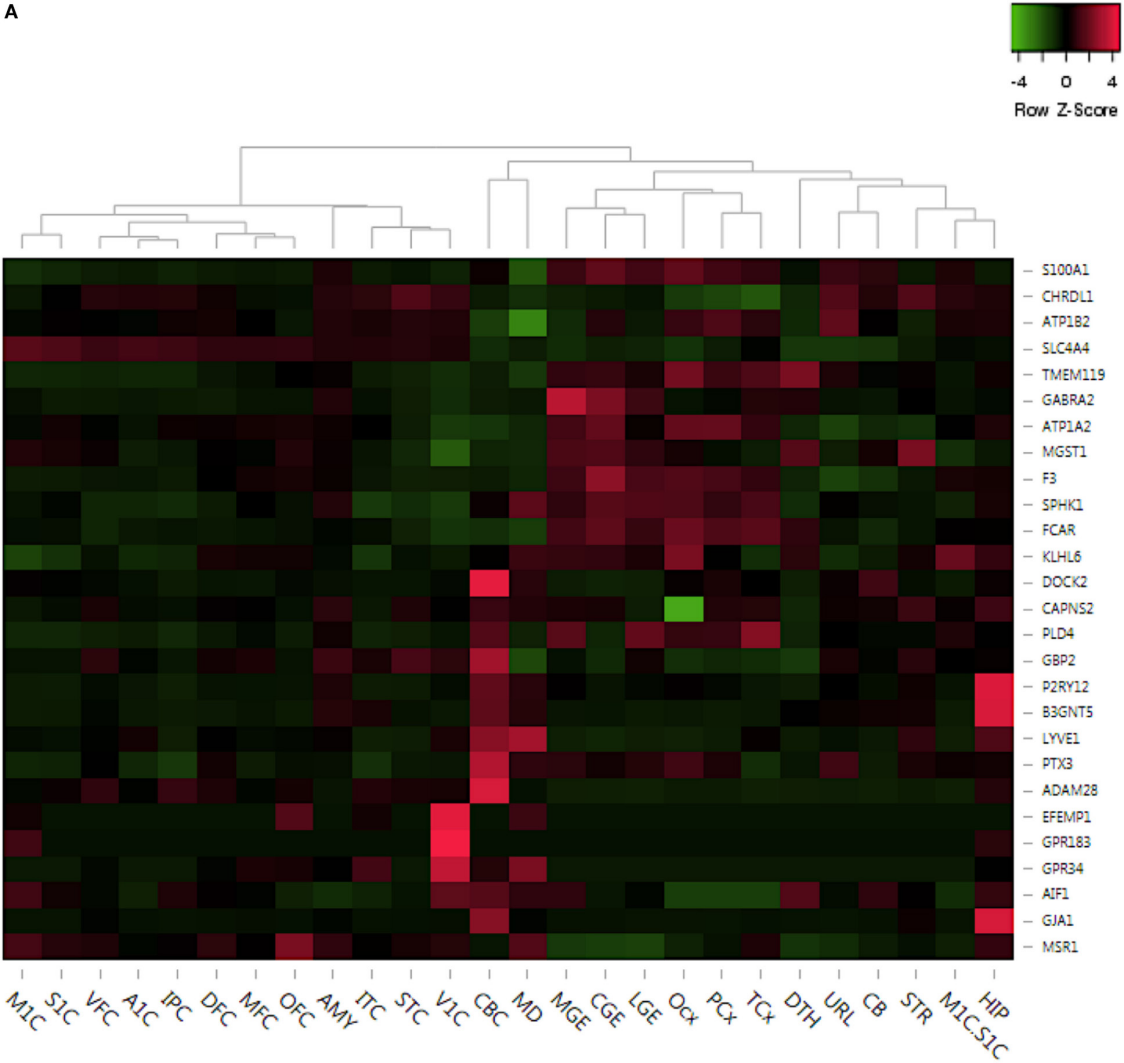

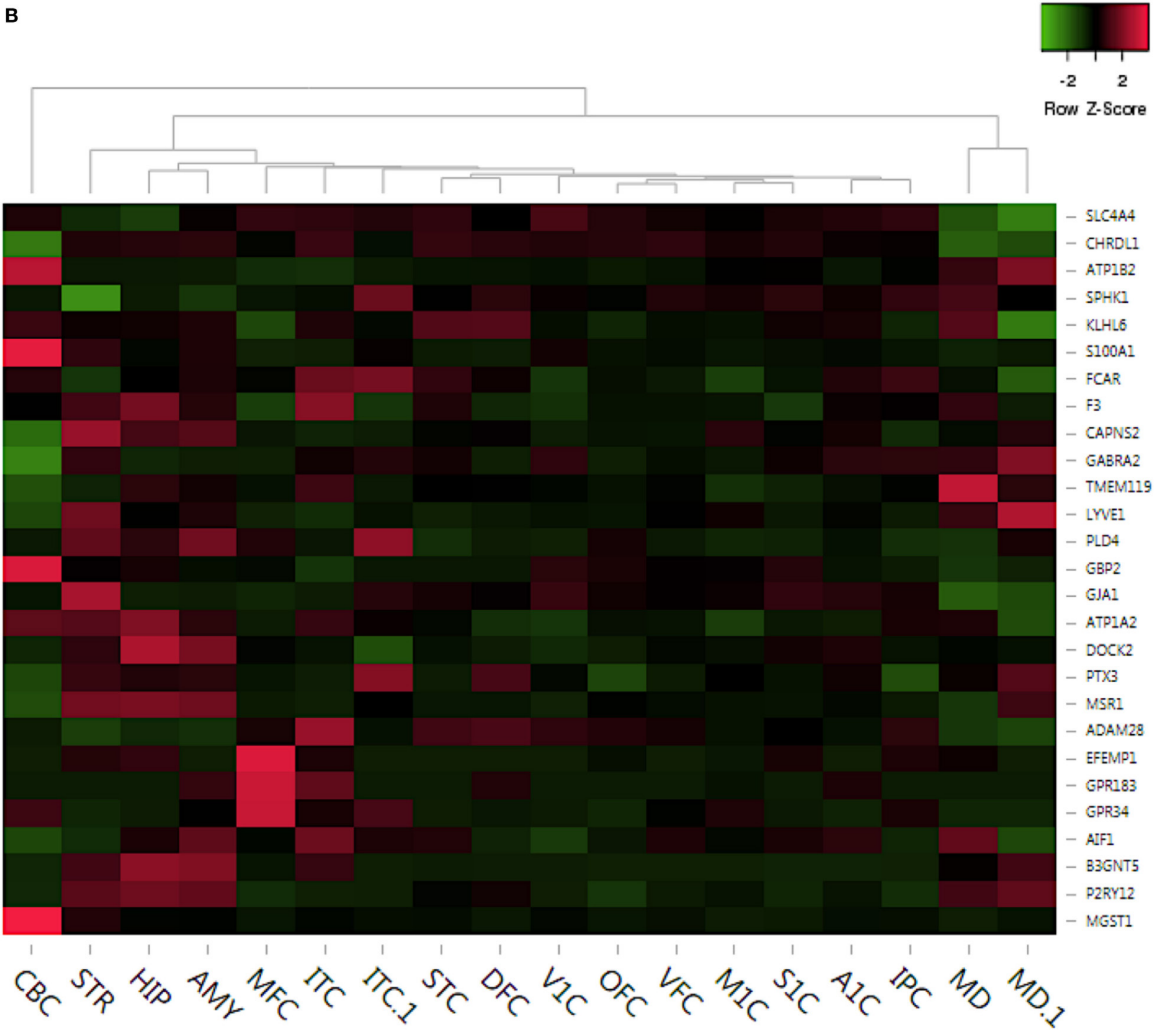
Delineation of top dysregulated gene expression profiles across crucial brain anatomic regions associated to neurodevelopment. **(A)** Prenatal region-wise expression of the top upregulated and downregulated genes. **(B)** Postnatal region-wise expression of the top upregulated and downregulated genes. Abbreviations: DFC, dorsolateral prefrontal cortex; VFC, ventrolateral prefrontal cortex; MFC, anterior (rostral) cingulate (medial frontal cortex); OFC, orbital frontal cortex; M1C, primary motor cortex (M1); S1C, primary somatosensory cortex (S1); IPC, posteroinferior (ventral) parietal cortex; A1C, primary auditory cortex (core); STC, posterior (caudal) superior temporal cortex (Tac); ITC, inferolateral temporal cortex (Tev); V1C, primary visual cortex (striate cortex, V1); HIP, hippocampus (hippocampal formation); AMY, amygdaloid complex; STR, striatum; MD, mediodorsal nucleus of thalamus; CBC, cerebellar cortex.

In the prenatal brain, the expression levels of known biomarker genes like *PLD4, SPHK1* were high in regions of importance like caudal ganglionic eminence, medial ganglionic eminence, lateral ganglionic eminence and lower in regions like posteroinferior parietal cortex, inferolateral temporal cortex, medial frontal cortex (MFC) (Figure 5A). However, *GPCR* associated genes like *GPR183* and *GPR34*, and EFEMP1 were seen to be highly expressed only in the primary visual cortex (V1C) region in the prenatal brain (Figure 5A). To highlight, *EFEMP1* mutations are associated with Doyne honeycomb retinal dystrophy, an autosomal dominant disorder in which there are drusen (lipid) deposits in the macula eventually leading to vision loss (64, 65). In the postnatal brain, genes such as *GBP2*, astrocyte marker *S100A1* and *MGST1* are highly expressed in the cerebellar cortex region, while genes like *GPR183, EFEMP1*, and *GPR34* are only expressed in MFC (Figure 5B).

In summary, this analysis revealed consistent high expression of 14 out of 27 microglial markers in regions like hippocampus (HIP), amygdala (AMY), and striatum (STR) during postnatal development (Figure 5B). However, all of these 14 genes are low in expression throughout prenatal development (Figure 5A). Interestingly, amygdaloid complex (AMY) of the prenatal brain showed very low expression of all 27 genes, whereas the same region showed pronounced expression of numerous genes, like *P2RY12, DOCK2, MSR1, B3GNT5, AIF1*, etc. during postnatal development (Figure 5B). Overall, this analysis has provided region-based developmental tracking of microglial expression dynamics in the human brain.

### Age-Specific Correlation of Microglial Signatures During Brain Development

Ontogeny, renewal, and maintenance of microglial population have long been debated. In this direction, we decided to correlate region-specific expression of microglial markers to developmental stages of the human brain. The ABA developmental transcriptome data yielded a humungous gene pool constituting age-wise microglial marker expression specifically in AMY, HIP and STR regions. We, further, focused on the gene regulation status of top 10 upregulated and downregulated gene sets across brain ages encompassing prenatal (8–37 pcw) and postnatal (1–40 years). Furthermore, we built correlation matrices between several important ages, including 13 and 37 pcw, marking the prenatal ages, and 1, 8, 15, 19, and 40 years indicating infant to the adult brain.

The correlation matrices with a correlation coefficient scale (−1, 1) for all three brain regions showed positive correlation at varying degrees. The representative correlogram for prenatal stages of *HIP* showed moderately positive correlation (*R* = 0.65) between 13 and 37 pcw and aged brain stage of 40 years (*R* = 0.70) (Figure S2 in Supplementary Material). Interestingly, a very strong correlation was also noticed between the early adult ages of 8 years and later ages like 18 and 40 years (*R* = 0.90) in the *HIP* region. Respective correlogram for *STR* displayed increasing correlation (0.77) between 13 and 37 pcw and all adult ages, and notably, highest correlation score was observed between 19 and 40 years of age (*R* = 0.97) (Figure S2 in Supplementary Material). In case of *AMY*, we noticed very strong correlation between the postnatal ages of 1, 5, 15, and 40 years (*R* = 0.88). However, a moderate correlation was observed among 13 and 37 pcw (*R* = 0.67), and 13 pcw and 40 years stages in case of *AMY* region (Figure S2 in Supplementary Material).

#### Upregulated Genes in Age-Wise Distribution

**Figure 6:**
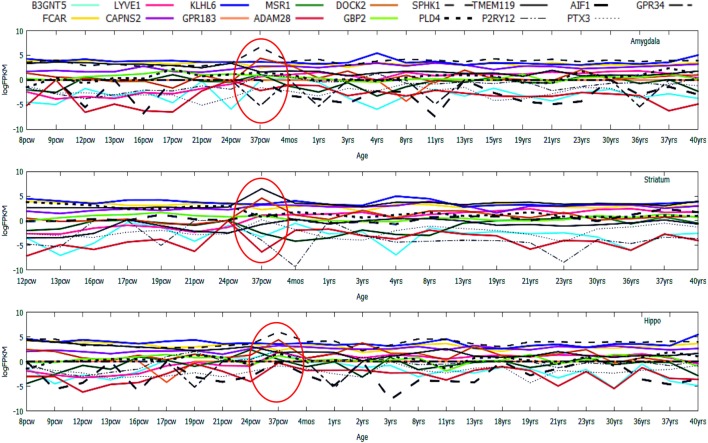
Age-wise expression dynamics of highly upregulated genes across microglia-enriched regions of the human brain. Gene expression profiling of top 10 upregulated genes and known expressed biomarkers (17) in the developing/degenerating brain including prenatal stages like 9 pcw fetus and postnatal brain stages like 3 years to a grown adult 40-year-old brain.

In the *HIP*, known biomarker genes like *AIF1* was found to be consistently high (log_2_FPKM ~ 5.0), while the expression levels of *SPHK1* and *GBP2* remained low (log_2_FPKM < 0). In *STR, GBP2* was highly expressed, while *AIF1* and *SPHK1* were low in expression (log_2_FPKM < 0). In *AMY, AIF1* and *SPHK1* showed consistently high expression (log_2_FPKM ~ 3.5, 3.0), while GBP2 remained low in expression throughout prenatal to postnatal brain developmental stages (log_2_FPKM < 0). In case of the top upregulated genes (10), variation in gene expression was strongly evident (Figure 6).

Among the top regulated genes, Lymphatic Vessel Endothelial Hyaluronan Receptor 1 (*LYVE1*), a type I integral membrane glycoprotein was found to be consistently repressed till the prenatal stages, followed by gradual increment in expression during postnatal stages (after 37 pcw) with minor inconsistencies in all the three brain regions (Figure 6). A supporting study from mice also showed similarity in the variation of *LYVE1* expression profiles in hippocampal and cerebellar regions (19). Consistent high expression of *FCAR* and *KLHL6* throughout pre- and postnatal development was distinct in all three aforementioned regions. The most inconsistently expressed genes across all the three regions included *DOCK2* and *GPR183* exhibiting a rapid decrease in expression at various ages like 12 pcw, 17 pcw, 24 pcw, 8 years, 21 years, 30 years, and 37 years suggestive of their dynamic roles in brain aging.

#### Downregulated Genes in Age-Wise Distribution

**Figure 7:**
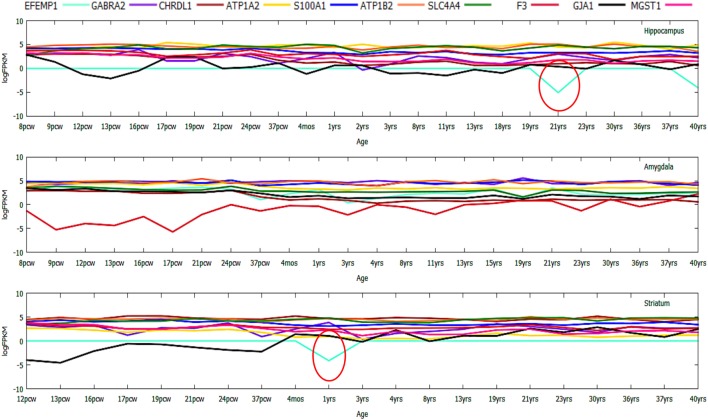
Gene expression profiling of top 10 downregulated genes during prenatal to postnatal brain developmental stages displaying numerous inconsistently or dynamically expressed genes like *DOCK2*.

**Figure 8:**
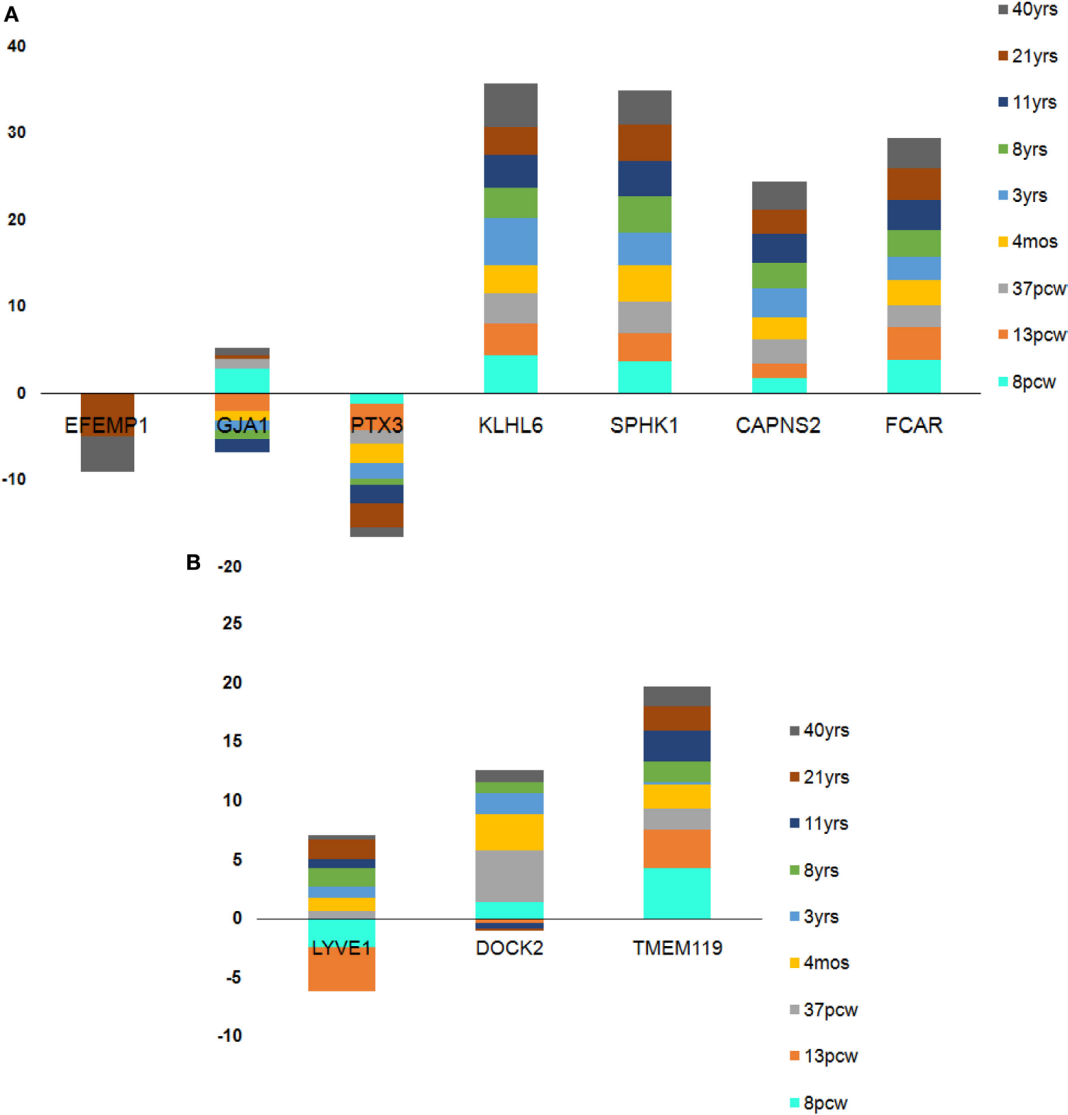
Age-wise dynamics yielded crucial microglia-specific signatures. **(A)** Seven genes found to be consistently high in expression from pre- to prenatal brain developmental stages. **(B)** Three genes found to be dynamically expressed with sharp dips at the transition stage of 37 postconception week (pcw) except in case of *DOCK2* which was inconsistently expressed throughout pre- and postnatal stages. Each stack in the stacked bar plot represents a single brain age whose height denotes the amount of expression.

In case of the top downregulated gene set (10), a uniform pattern of expression was observed. In the *HIP*, all the genes were found to be consistently high in expression (log_2_FPKM ~ 4.5) throughout all the age groups excluding two inconsistently expressed genes, *GJA1* and *EFEMP1* (Figure 7). We also found that *EFEMP1* showed unaltered expression till the age of 19 years, followed by a sharp dip in expression at the ages of 21 and 40 years. On the other hand, *GJA1* showed prominent inconsistency with a gradual decline toward 13 pcw, increase at 17 pcw, sharp dip at the age of 4 months and finally, remained unaltered in the rest of the postnatal-adult ages. In *AMY*, all the genes showed consistently high expression (log_2_FPKM ~ 4.0), which then gradually decreased toward the postnatal stages (log_2_FPKM ~ 3.0) excluding F3 gene. Surprisingly, expression of *F3* was seen to be significantly low (log_2_FPKM < −2) during prenatal stages, with inconsistency in expression during postnatal ages (Figure 7). In *STR*, all genes except *EFEMP1, GJA1*, and *GABRA2* were seen to be continually expressed at a high level throughout all age groups. Especially, *GABRA2* showed sharp dips at 17 pcw, 37 pcw, and 3 years whereas, *GJA1* remained repressed (log_2_FPKM < 0) with gradual increment in expression during the postnatal stages (log_2_FPKM ~ 1.0) (Figure 7). Early pieces of evidences from *GJA1*/Connexin 43 null and conditional knock-out mouse brains revealed its strong regulatory role in overall brain development (66). The age-wise expression map (DDM) represented a set of gene fingerprints specific to microglia which consisted of seven genes, namely, *EFEMP1, GJA1, PTX3, KLHL6, SPHK1, FCAR*, and *CAPNS2* (Figure 8A). This gene set showed consistently altered expression (both up and downregulated) in all three microglia-dense regions, across the human brain development. To highlight, DDM also revealed three most dynamically regulated genes namely, *DOCK2, LYVE1*, and *TMEM119* demonstrating an irregular pattern of expression across the brain ages (Figure 8B). These gene signatures can be further explored *in vitro* to elucidate their specific roles in microgliogenesis.

### *In Vitro* Validation of Microglia-Specific Biomarkers in the Cellular Model of Microglia

**Figure 9:**
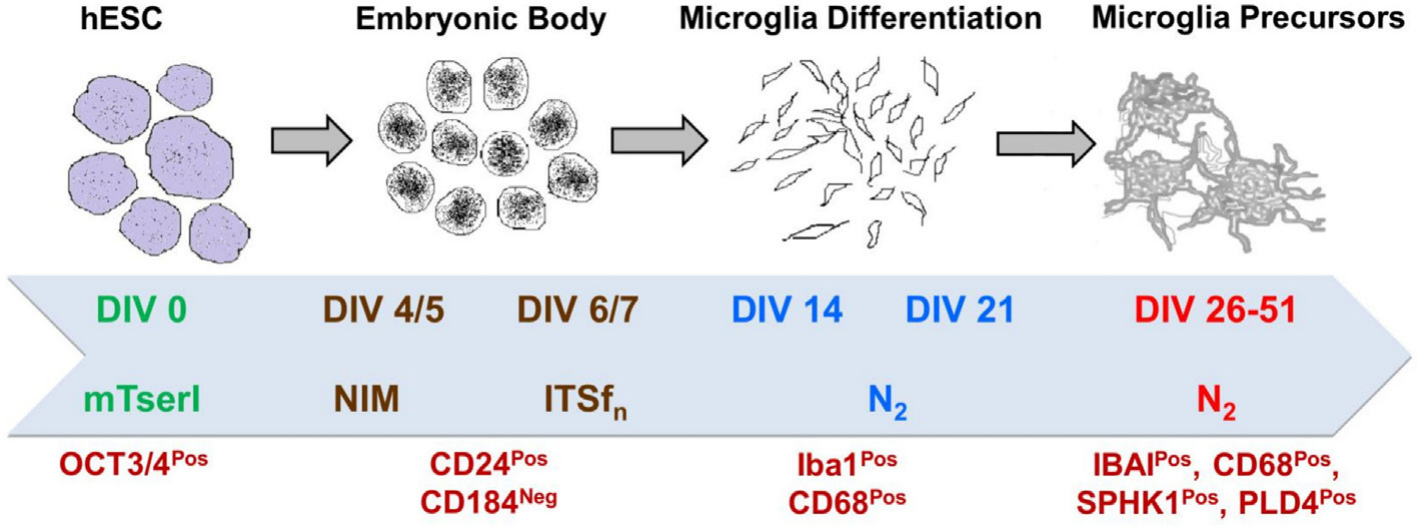
Brief scheme demonstrating the protocol of directed differentiation of hESCs into human microglia which is characterized by several stage-specific markers.

To validate the emergence of microglia-specific biomarkers during brain development, we have established a cellular model of human microglia using an *in-house* standardized method. To generate human microglial precursors *in vitro*, hESCs were induced for microglial commitment using a long-term culture platform, which could be maintained for a period of 4 to 6 weeks. To authenticate the generation of microglial precursors *in vitro*, we have evaluated the stage-specific lineage commitment that comprised of (i) formation of EBs, (ii) neural induction, and (iii) generation of early and late microglial precursors using specific biomarkers. Details of the culture stages have been explained in the scheme (Figure 9). Interestingly, the *in silico* age dynamics study has projected *SPHK1* as a key marker to be consistently upregulated at 37 pcw, specifically in *AMY* and *HIP* (Figure 6). It is already evident that *SPHK1* plays crucial roles in microglia-dependent neuroinflammatory cascades. Additionally, we also found *PTX3*, which is another marker of microglia involved in regulation of phagocytic activity to be consistently downregulated across DDM of the human brain, specifically in *AMY* and *HIP* (Figure 6). Based on these findings, we further tried to elucidate expression of *SPHK1* and *PTX3* in long-term culture of human microglia.

**Figure 10:**
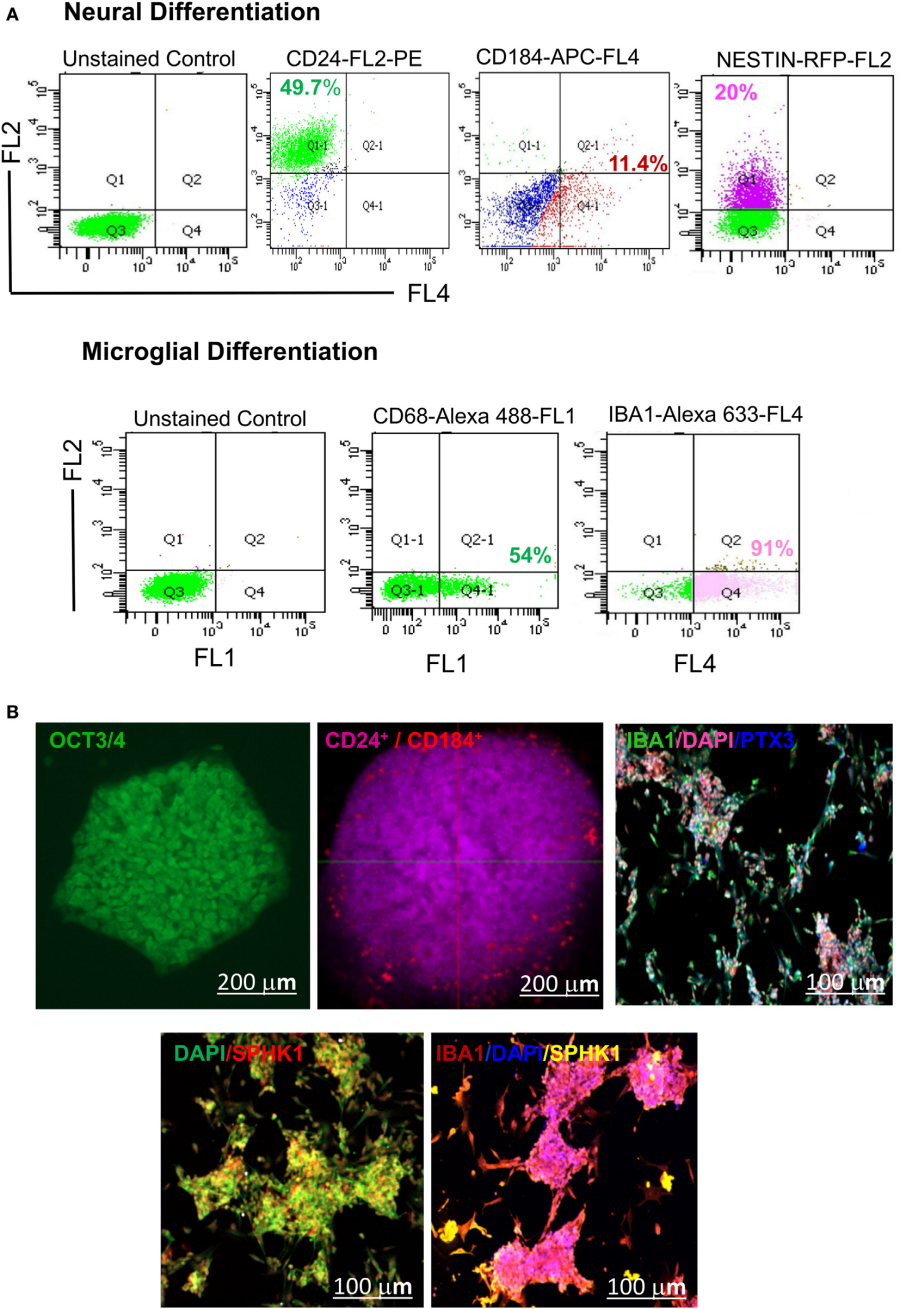
Identification of hESC-derived microglia precursors *in vitro* using different stage-specific markers. **(A)** Flow cytometry based analysis demonstrated characterization of microglia induction and differentiation of microglial precursors. The analysis indicated differentiation of hESCs into embryonic bodies (EB, 5 DIV), as shown by expression of ~49.7% *CD24***^Pos^** and 11.4% *CD184***^Pos^** population. This is followed by neural induction as shown by 20% of *NESTIN***^Pos^** cells. Neurally induced cells further showed the generation of a distinct population having 54% of *CD68***^Pos^** cells and 91% *IBA1***^Pos^** cells. **(B)** Confocal imaging of hESC-derived EBs showed expression of *CD24* and *CD184 in vitro* at 5 DIV. The emergence of mature microglial precursors could be detected on 34DIV, as shown by *IBA1, SPHK1*, and *PTX3* expression. DAPI = nuclear marker, DIV = days *in vitro*. The scale bar represents size in micrometers.

Our results have demonstrated the appearance of 3D cellular aggregates on fifth DIV with differential expression of *CD24* and *CD184* in both flow cytometric and confocal imaging analysis (Figure 10). These cellular aggregates (EBs) were further induced to form early microglial precursors as evident by expression of *CD68* and *AIF1/IBA1* on 21DIV as shown in the flow cytometry micrographs (Figure 10A). These precursors later gave rise to microglial structures on 34DIV with distinct expression of two mature microglial markers *SPHK1* and *PTX3*, as evident in immunofluorescence-based studies (Figure 10B). This study has explored the optimal microenvironment for establishing cellular model mimicking human microglia and the associated important biomarkers.

## Discussion

Albeit microglia constitute approximately 10% of the total cell population in the CNS (67), there are major lacunae in the knowledge regarding their expression across human brain ages and regions (68). Many critical questions involving their ontogeny, localization, and self-renewal still need to be addressed. Discovery of novel microglial biomarkers and elucidation of their specific roles in brain developmental processes will shed light on the onset of neurological disease and progression.

Studies involving human microglia-derived transcriptomics and proteomics are still in its infancy, and given the low scope of availability of healthy/diseased human brain tissue, it is imperative to perform high-throughput data analysis to be able to identify and narrow down crucial microglia signatures (33). In this direction, using available tissue based transcriptomics (69) as well as induced microglia microarray data generated from pluripotent stem cells (25), we analyzed the differentially regulated gene sets pertinent to microglia biology and homeostasis, which resulted in a novel catalog of microglial biomarkers. Further using GSEA on the ascertained list, we have identified different clusters of gene sets specific to human microglia, which revealed several crucial clusters such as metalloproteinases, GPCRs, proteoglycans, immunoeffectors, gap junction family genes, and synaptosomal receptors, etc. These prominent gene clusters explained multidimensional roles and biological activity of microglia. This GSEA map acted as a primer for further downstream analyses. Correlation analysis further explained the differential expression behavior of many genes between the human brain cell types and induced microglia data sets, *viz*., *primary* and *secondary* data sets, respectively. Another interesting inference indicates that there might be different gene subsets required for maintaining microglial homeostasis and activity in the human brain, while enforced microglial differentiation *in vitro* requires an exclusive set of gene regulators. To understand the protein–protein interactions among the shortlisted candidates, we constructed a primary protein neighborhood network and performed pathway analysis. The study highlights some novel pathways associated with microgliasuch as Glycosaminoglycan metabolism, RAP1, Phospholipase C/D, *PPAR*, Fatty acid CoA signaling pathways. Upon assessment of the DDM across brain age groups, we have identified the top 20 altered genes as molecular signatures to developmentally track microglia in fetal, infant, child, adolescent, and adult brains.

Both GSEA and correlation analysis pointed out three possible hypotheses which might play roles in microglia-NPC crosstalk. The first hypothesis suggests that enhanced expression of *PTGS1/2* is linked to *EP2* receptors in microglia and might be the key to crosstalk with the qNSCs that express specifically *PGE2*. The second hypothesis discusses the interaction between microglia and qNSCs through plausible crosstalk between *DOCK2* and S1P, as evidence suggests qNSCs specifically express S1P as biomarker and microglia express SPHK1, *DOCK2*, and *S1PR1/5* (S1P receptor 1/5). The third hypothesis suggests that *GPR183* acts a conditioned rheostat for NSC quiescence and activity in *NOTCH1* dependent pathway. Since differential expression analysis showed high expression of *GPR183* in microglia, and *NOTCH1* regulates self-renewal of hippocampal NSCs, there can be possible molecular crosstalk (70, 71).

Studies from past few decades have indicated both specific and context-dependent localization and activity of microglia during neuroinflammatory circumstances (18, 39). However, hardly any study exists which could track their activity throughout brain development. Another major roadblock that is faced is the unavailability of human brain cells. To address such issues, we established a cellular model of the human microglial cell population and deciphered the expression of existing biomarkers, especially *CD68, AIF1 (IBA1), SPHK1*, and *PTX3*. Earlier findings have indicated that SPHK1 is found to be abundantly expressed in several rat brain cell types, including neurons in the hippocampus, cerebellar granule cells, and astrocytes and also in the amoeboid microglial cells of the corpus callosum of the postnatal brain (72). The *in silico* data strongly corroborated with this existing evidence, which suggested that *SPHK1* expression is significantly upregulated in microglia-dense regions, namely hippocampus and amygdala (Figure 6). The *in vitro* long-term culture study also supported these findings and suggested that human microglia precursors from post 26DIV showed consistent expression of *SPHK1* in late microglial precursors (Figure 10B). The results have also represented another unique marker, *PTX3* in the in cultured human microglial precursor cells, which is known to regulate the phagocytic activity of microglia (44). This observation supported the DDM data which has shown *PTX3* to be consistently downregulated across different ages in HIP and AMY of healthy brains. In summary, our *in vitro* study has validated the emergence of the microglial signatures in hESC-derived human microglial precursors and also represented a robust human microglial model for future investigations involving microglial biology and activity.

Microglia has been associated with onset, aggravation, and progression of neurodegenerative diseases like Alzheimer’s, Dementia, and Glioma (73–76). This is primarily due to dysregulated microglial activity during development as well as disease leading to severe neurocognitive impairments and neuronal dysfunction. Overall, this study has unraveled fundamental information underlying the labyrinthine molecular circuitry in microglia functioning, physiology, homeostasis, and genetics across brain developmental stages and regions.

## Conclusion

Our study has introduced a novel integrative biology approach for tracking human microglia signatures across 25 developmental stages spanning fetal, infant, adolescence, and adult, and delineated their localized expression in microglia-dense regions. The *in vitro* data have also validated the expression of some of the biomarkers in human microglial precursors. Precisely, tracking of microglia transcriptomic dynamics based on brain anatomy and age could establish an essential DDM comprising highly altered gene clusters. This map can be further utilized to develop set of putative prognostic markers in accordance with the age of the human brain. One of the interesting readouts of the DDM was the dynamic expression associated with 37 pcw, i.e., a transition period between prenatal and postnatal brain. To highlight, the study has led to the discovery of microglia age-specific gene signatures, including *EFEMP1, GJA1, PTX3, KLHL6, SPHK1, FCAR*, and *CAPNS2*, which can be further explored for understanding their roles in microglial biology in the human brain.

## Future Perspectives

In essence, this study has established a repertoire of microglia-specific signatures involved in the developing human brain. Characterization of mechanistic roles of these gene fingerprints in experimental models would further bridge the gap in understanding the molecular mechanisms involved in microglia ontogeny, renewal, and maintenance. Age-wise expression profiling of these novel signatures (DDM) in both pre- and postnatal brain stages can act as guidance cues for designing future prognostic and diagnostic biomarkers against neurodegenerative diseases. Finally, the data associated with age and region-specificity can pave the way to personalized medicine interventions for debilitating neurological diseases.

## Author Contributions

RA: performed analyses, computations, and graphics. RA and SP: designed the methodological approach and wrote the manuscript. SP and SS: read, critically analyzed, and approved the final manuscript.

## Conflict of Interest Statement

The authors declare that the research was conducted in the absence of any commercial or financial relationships that could be construed as a potential conflict of interest.

## Footnotes

^1^http://www.broadinstitute.org/gsea (Accessed: July 17, 2017).

^2^http://www.broadinstitute.org/gsea/msigdb (Accessed: July 17, 2017).

^3^www.geneontology.org (Accessed: July 17, 2017).

## Supplementary Material

Figure S1 Donut plot showing various protein classes associated with the differentially expressed gene set or 3,290 genes.

Figure S2 Correlation matrix/correlogram showing varying correlation ranging from very weak to very strong (0.0–0.99) among significant brain ages within three microglia-enriched regions, namely AMY, HIP, and STR. The color bar depicts the range of correlation coefficient (−1, 1) and in each correlogram, the colored slice in the pie increases in size and intensity in accordance with the correlation coefficient value.
