# Supplementary figures and images for: Deconvolution of Human Brain Cell Type Transcriptomes Unraveled Microglia-Specific Potential Biomarkers
Source: Front Neurol. 2018 Apr 26;9:266. doi: 10.3389/fneur.2018.00266 (PMC5932158; doi:10.3389/fneur.2018.00266)

Supplementary figure 1.

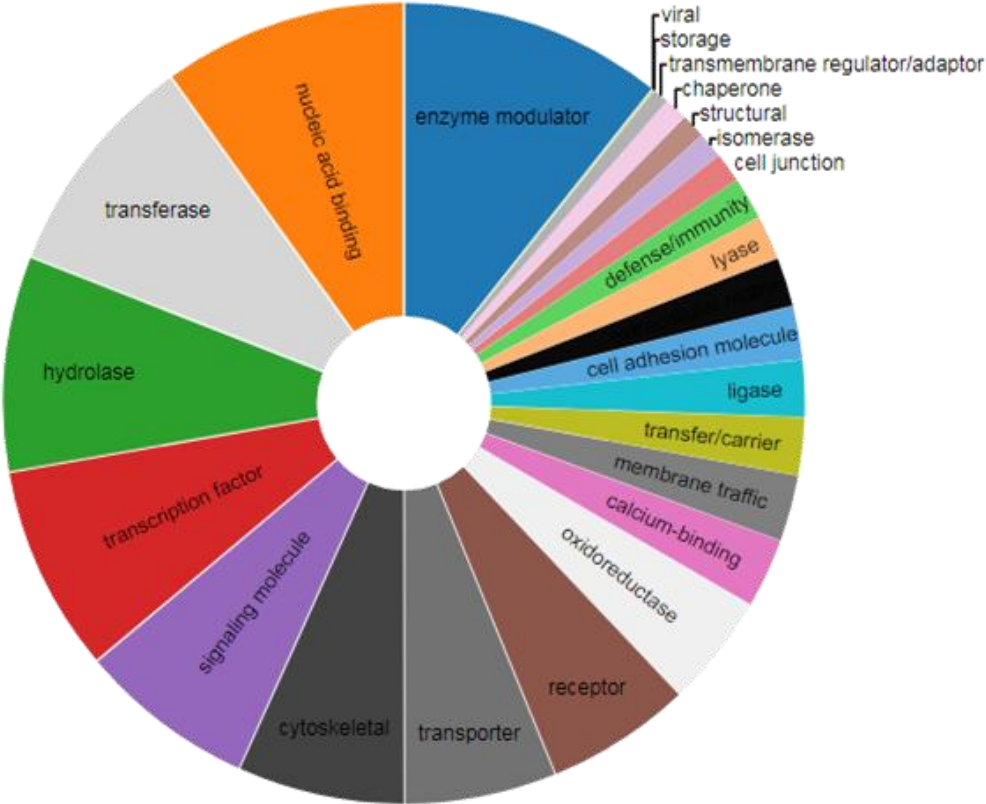

Supplementary figure 2.

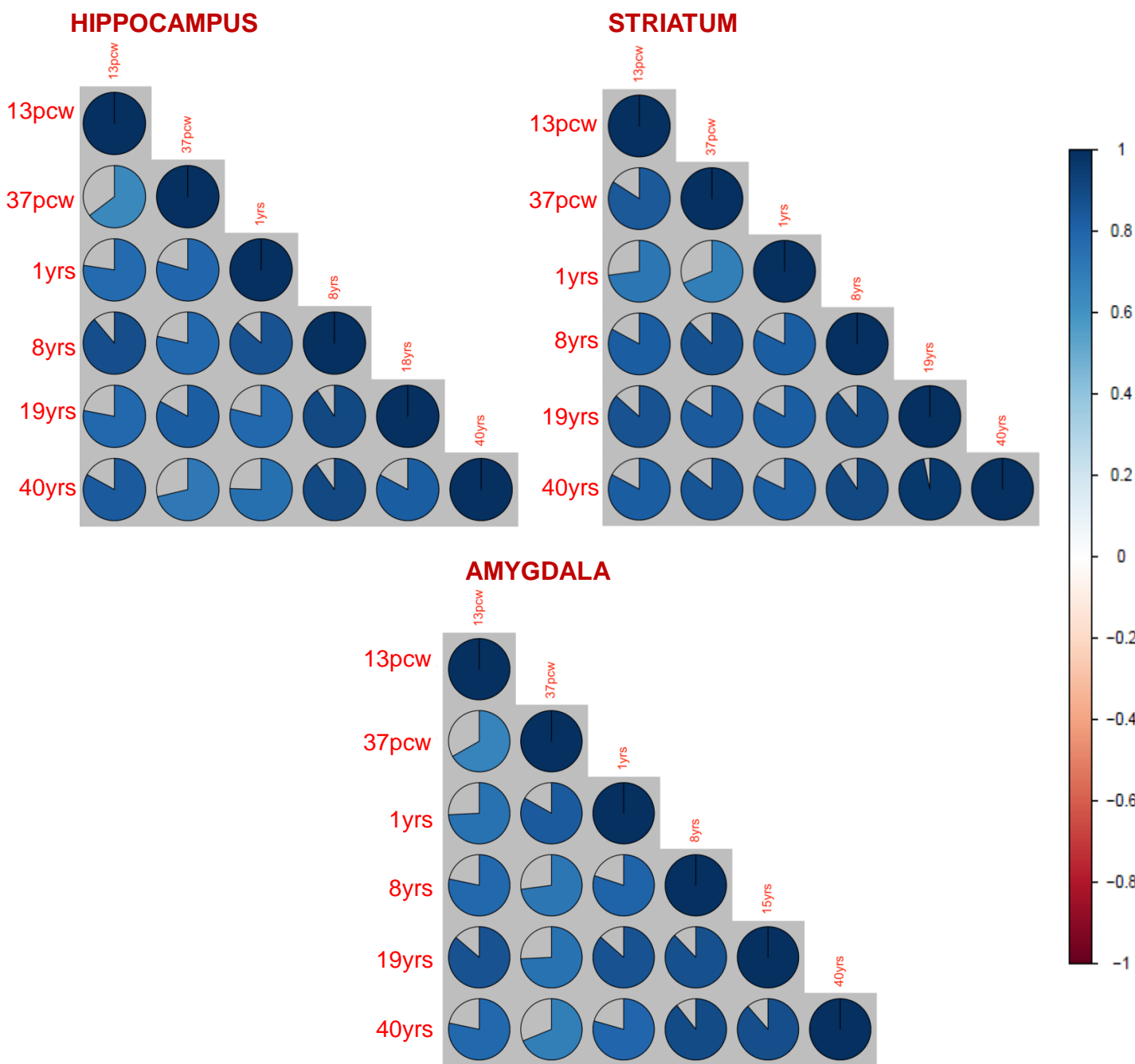

Supplement: Figure S1 — Donut plot showing various protein classes associated with the differentially expressed gene set or 3,290 genes. [file image_1.pdf]
