# Supplementary material for: Deconvolution of Human Brain Cell Type Transcriptomes Unraveled Microglia-Specific Potential Biomarkers
Source: Front Neurol. 2018 Apr 26;9:266. doi: 10.3389/fneur.2018.00266 (PMC5932158; doi:10.3389/fneur.2018.00266)
Supplement: Supplementary file 2 [file data_sheet_1.pdf]

Supplemental Information

Confocal Imaging:

Cells from each stages of microglial differentiation were grown on pre-treated German glass cover slips (GG-18-1.5-pre, NeuVitro, Vancouver, USA). Prior to fixing, they were gently washed 3X with PBS-T (PBS containing 0.1% Triton-X100) for 10 min, followed by two hours of incubation in blocking solution (PBS-T with 5% goat serum). Both conjugated and unconjugated primary antibodies were prepared in the above blocking solution. Following the blocking step, coverslips were incubated overnight 4°C with the primary antibodies. After the overnight incubation, the glass coverslips were washed again 3X in PBS-T for 15 min, and further incubated with respective secondary antibodies for 2 hours at room temperature (RT). The coverslips were washed then 3X for 10 min in PBS-T following incubation at RT and counterstained with ProLong™ Diamond Antifade Mountant containing DAPI for 15 min at RT. Finally the coverslips were washed for 2X in PBS and kept in dark prior to imaging. The confocal images were acquired in Zeiss LSM 710 and analysed using ZEN 2012 SP1 software (Zeiss, Thornwood, NY). Please see the antibody list below for reference (Supplementary table 1).

Flow cytometry analysis

Cultured cells from different stages of microglia induction and differentiation were enzymatically harvested by Accutase treatment for 5 min at 37°C and then gently scrapped with cell scraper (Sigma-Aldrich). Cells were then washed twice using a customized staining buffer that contained 1x PBS with 0.2% bovine serum albumin and 0.09% sodium azide. Cells were pelleted in 5810R Eppendorf centrifuge at a speed of 1600 rpm for 5 mins. These cell pellets were re-suspended in 200 µl of their respective medium containing the required ratio of fluorescence-conjugated antibodies as per the manufacturer's instructions and were incubated on ice for 45 min in a dark place. Following incubation with unconjugated primary antibodies, the cells were washed twice using the above staining buffer and again pelleted using similar centrifugation step. Cell pellets were further resuspended with florochrome-conjugated secondary antibodies and incubated on ice for 1 hour. Prior to analysis, cells were washed, re-pelleted and finally resuspended in the staining buffer. Isotype controls or secondary antibodies only were used to quantitate the baseline background. Data was acquired using a BD Biosciences ARIA-III workstation and analyzed using BD FACS Diva™ software.

Supplementary Table 1. List of Antibodies used for flow cytometry and immunofluorescence analyses.

Name	Host	Vendor	Cat. No.
**CD184-APC**	**Mouse**	BD Pharmingen	555976
**CD24-PE**	**Mouse**	BD Pharmingen	555428
**SPHK1**	**Mouse**	Santa Cruz Biotechnology, Inc.	sc-365401

**Oct3/4-PE**	**Mouse**	BD Pharmingen	560186
**PTX3**	**Mouse**	R&D Systems	PP-PPJ0069-00
**IBA1**	**Rabbit**	Wako	019-19741
**Anti-mouse IgM-RRX**	**Goat**	Jackson Immunoresearch	115-296-020
**Anti-rabbit IgG-Alexa-633**	**Goat**	Invitrogen	A21070
**Anti-mouse IgG-Alexa-594**	**Goat**	Invitrogen	A11012

Supplementary Table 2. The mapped GO ID to corresponding GO Molecular function /Biological process names.

**GO ID**	**GO process**	**Adj_pvalue**
**GO:0004872**	receptor activity	3.70E-08
**GO:0004871**	signaling transducer activity	5.82E-08
**GO:0010644**	cell communication by electrical coupling	1.02E-07
**GO:0038023**	signaling receptor activity	1.22E-07
**GO:0051270**	regulation of cellular component movement	4.12E-07
**GO:0008284**	positive regulation of cell proliferation	5.92E-07
**GO:0034220**	ion transmembrane transport	6.32E-07
**GO:0044057**	regulation of system process	6.70E-07
**GO:0009605**	response to external stimulus	1.15E-06
**GO:0071310**	cellular response to organic substance	1.30E-06
**GO:0051050**	positive regulation of transport	1.50E-06

**GO:0098660**	inorganic ion transmembrane transport	1.51E-06
**GO:0086065**	cell communication involved in cardiac conduction	1.73E-06
**GO:0010959**	regulation of metal ion transport	1.77E-06
**GO:0009719**	response to endogenous stimulus	2.29E-06
**GO:0055085**	transmembrane transport	2.34E-06
**GO:0042127**	regulation of cell proliferation	2.88E-06
**GO:0002376**	Immune system process	4.30E-06
